# Parthenolide induces apoptosis via TNFRSF10B and PMAIP1 pathways in human lung cancer cells

**DOI:** 10.1186/1756-9966-33-3

**Published:** 2014-01-06

**Authors:** Xiaofei Zhao, Xiangguo Liu, Ling Su

**Affiliations:** 1 Shandong Provincial Key Laboratory of Animal Cells and Developmental Biology, School of Life Sciences, Shandong University, Room 103, South Building, 27 Shanda South RD, Jinan 250100, P.R. China

**Keywords:** Parthenolide, TNFRSF10B, CFLAR, PMAIP1, Endoplasmic reticulum stress, DDIT3

## Abstract

**Background:**

Parthenolide (PTL) is a sesquiterpene lactone which can induce apoptosis in cancer cells and eradicate cancer stem cells such as leukemia stem cells, prostate tumor-initiating cells and so on. However, the mechanism remains largely unclear.

**Methods:**

Lung cancer cells were treated with parthenolide and the cell lysates were prepared to detect the given proteins by Western Blot analysis, and the cell survival was assayed by SRB and MTT assay. Cell cycle was evaluated by DNA flow cytometry analysis. TNFRSF10B, PMAIP1, ATF4 and DDIT3 genes were knocked down by siRNA technique. Apoptosis was evaluated by using Annexin V-FITC/PI staining and flow cytometry analysis.

**Results:**

Parthenolide (PTL) induces apoptosis and cell cycle arrest in human lung cancer cells. Moreover, PTL treatment in NSCLC cells increases expression of TNFRSF10B/DR5 and PMAIP1/NOXA. Silencing of TNFRSF10B or PMAIP1 or overexpression of CFLAR /c-FLIP (long form) could protect cells from PTL-induced apoptosis. Furthermore, PTL could increase the levels of endoplasmic reticulum stress hallmarks such as ERN1, HSPA5, p-EIF2A, ATF4 and DDIT3. Knockdown of ATF4 and DDIT3 abrogated PTL-induced apoptosis, which suggested that PTL induced apoptosis in NSCLC cells through activation of endoplasmic reticulum stress pathway. More importantly, we found that ATF4, DDIT3, TNFRSF10B and PMAIP1 were up-regulated more intensively, while CFLAR and MCL1 were down-regulated more dramatically by PTL in A549/shCDH1 cells than that in control cells, suggesting that PTL preferred to kill cancer stem cell-like cells by activating more intensive ER stress response in cancer stem cell-like cells.

**Conclusion:**

We showed that parthenolide not only triggered extrinsic apoptosis by up-regulating TNFRSF10B and down-regulating CFLAR, but also induced intrinsic apoptosis through increasing the expression of PMAIP1 and decreasing the level of MCL1 in NSCLC cells. In addition, parthenolide triggered stronger ER stress response in cancer stem cell-like cells which leads to its preference in apoptotic induction. In summary, PTL induces apoptosis in NSCLC cells by activating endoplasmic reticulum stress response.

## Background

Parthenolide is a sesquiterpene lactone derived from the plant feverfew. It is used to treat inflammation due to its ability of inhibiting NF-κB activity [1]. Parthenolide has also been reported to play other roles such as promoting cellular differentiation, causing cells to exit cell cycle and inducing apoptosis [2,3]. Its pro-apoptotic effect on cancer cells is known to trigger the intrinsic apoptotic pathway which includes elevated levels of intracellular reactive oxygen species (ROS) and alteration of BCL2 family proteins [4-6]. What’s more, recent studies have revealed that PTL could selectively eradicate acute myelogenous leukemia stem and progenitor cells [7]. It is also demonstrated that PTL could preferentially inhibit breast cancer stem-like cells [8], but the molecular mechanism was still unclear.

There are two major pathways contributing to apoptotic signaling: the extrinsic death receptor pathway and the intrinsic mitochondrial pathway [9]. Death receptor 5 (TNFRSF10B) is a protein that belongs to tumor necrosis factor receptor (TNFR) superfamily [10]. It contains a cytoplasmic death domain (DD) which can recruit Fas-Associated Death Domain (FADD) and caspases to form the Death-Inducing Signal Complex (DISC) when the receptor is trimerized [11]. Subsequently, initiator caspases are activated and lead to the cleavage of downstream effectors. The activation of CASP8 can be regulated by FLICE-like inhibitor protein (CFLAR) which prevents recruitment of CASP8 to DISC [12,13]. Development of pro-apoptotic agonists has been focused on TNFRSF10B because of its target selectivity for malignant over normal cells [14,15].

The imbalance among the BCL2 family members which have been defined as either anti-apoptotic or pro-apoptotic is essential for the modulation of intrinsic pathway [16,17]. The BH3-only protein PMAIP1 is a p53 transcriptional target in response to DNA damage [18]. It has been reported to be involved in chemotherapeutic agent-induced apoptosis [19]. PMAIP1 can interact with MCL1 which is a pro-survival BCL2 protein, then displacing BCL2L11 from the MCL1/BCL2L11 complex and freeing BCL2L11 to trigger the intrinsic pathway [20]. This association can also promote proteasomal degradation of MCL1 to enhance the mitochondrial apoptosis [21].

Chemotherapy has been reported to induce ER stress response in cancer cells [22]. ER stress is usually caused by accumulation of misfolded or unfolded proteins in the ER lumen. When those proteins are not resolved, ER stress is prolonged to induce apoptosis [23,24].There are several mechanisms linking ER stress to apoptosis such as cleavage and activation of pro-CASP12 and activation of ASK1 [25]. Many studies have focused on the ER stress effector DDIT3, which is a downstream target of ATF4 [26]. DDIT3 is a bZIP-containing transcription factor that can target several apoptotic genes including TNFRSF10B and PMAIP1 [27]. The molecular mechanisms of ER stress-induced apoptosis still require further study.

Cancer stem cells have many similar aspects with stem cells. Those cells have the ability of self-renewal and differentiation, express typical markers of stem cells [28]. They are also considered to be the origin of cancer cells and are rather resistant to active drugs. Many reports have indicated that cancer stem cells are correlated with poor clinical prognosis [29,30]. So, targeting cancer stem cell may be a promising strategy for cancer therapy. PTL could preferentially inhibit cancer stem cells, but the molecular mechanism was still unclear.

In our study, we explored the mechanism signaling pathways involved in PTL-induced apoptosis in non-small cell lung cancer (NSCLC) cells and the role of ER stress in this process. We also found a potential mechanism why PTL would selectively eradicate cancer stem-like cells, which may have clinical implications in eradicating cancer stem cells eventually.

## Methods

### Antibodies and reagents

Parthenolide and PMAIP1 antibody were purchased from Calbiochem (Darmstadt, Germany). Briefly, parthenolide was dissolved in dimethyl sulfoxide (DMSO) at a concentration of 10 mmol/L, and the aliquots were stored at -20°C. Stock solutions were diluted to the desired concentrations with growth medium before use. The antibodies of TNFRSF10B and ACTB were purchased from Sigma-Aldrich (St. Louis, MO, USA). CDH1 and CFLAR antibodies were obtained from BD Biosciences (San Jose, CA, USA) and Alexis (San Diego, CA) respectively. Anti-CASP8, CASP9, HSPA5, MCL1, p-EIF2A, and PARP1 antibodies were purchased from Cell Signaling Technology (Danvers, MA, USA). CASP3 anti-body was obtained from Imgenex (San Diego, CA, USA). Antibodies of ATF4, DDIT3 were obtained from Santa Cruz (Santa Cruz, CA).

### Cell lines and cell culture

Human lung cancer cell lines were obtained from the American Type Culture Collection (Manassas, VA). Cells were gown in monolayer culture with RPMI 1640 medium containing 5% new born calf serum at 37°C in a humidified atmosphere consisting of 5% CO2 and 95% air. The A549/Ctrl, A549/CFLAR, H157/Ctrl, H157/CFLAR, A549/shCtrl and A549/shCDH1 stable cell lines are established earlier by infection with lentiviral production [31,32].

### Cell survival assay

Cells were seeded in 96-well plates and treated on the second day with the given concentration of PTL for another 48 hours and then subjected to SRB or MTT assay. For SRB assay, live cell number was estimated as described earlier [33]. After treatment, the medium was discarded firstly. In order to fix the adherent cells, 100 μ1 of cold trichloroacetic acid (10% (w/v)) were adding to each well and incubating at 4°C for at least 1 hour. The plates were then washed five times with deionized water and dried in the air. Each well were then added with 50 μ1 of SRB solution (0.4% w/v in 1% acetic acid) and incubated for 5 min at room temperature. The plates were washed five times with 1% acetic acid to remove unbound SRB and then air dried. The residual bound SRB was solubilized with 100 μ1 of 10 mM Tris base buffer (pH 10.5), and then read using a microtiter plate reader at 495 nm. The MTT assay was executed following the manufacturer’s protocol of Cell Proliferation Kit I (Roche Applied Science, Brandford, CT, USA). 20 μl MTT (5 mg/ml) were added to each sample and incubate at 37° for 4 h, then 100 μl solubilization solution were added. Cell viability was determined at 595 nm.

### Cell cycle analysis

Cell cycle was evaluated by DNA flow cytometry analysis. Cells were treated with different concentrations of PTL (0, 5, 10, 20 μM) for 24hours. After treatment, the cells were harvested and washed twice with ice PBS, then fixed in 70% ethanol at -20°C overnight. Before analysis, cells were washed again with ice PBS, incubated with PI (100 μg/ml) and RNase (50 μg/ml) in the dark for 30 min. Then samples were analyzed by FACScan flow cytometer (Becton Dickinson, San Jose, CA) [34].

### Western blot analysis

Whole cell protein lysates were prepared and analyzed by Western blot according to the protocol described previously [35]. Cells were harvested and rinsed with pro-cold PBS. Then cell extracts were lysed and centrifuged at 4°C for 15 minutes. Whole cell protein lysates (40 μg) were electrophoresed through 12% denaturing polyacrylamide slab gels and then transferred to a Hybond enhanced chemiluminescence (ECL) membrane by electroblotting. The proteins were probed with the appropriate primary antibodies and subsequently with secondary antibodies. The antibody binding was detected by the ECL system (Millipore, Billerica, MA, USA), according to the manufacturer’s protocol.

### siRNA transfection

siRNAs targeting sequences of TNFRSF10B, ATF4 and DDIT3 have been described previously and synthesized by GenePharma (Shanghai, China) [36]. The target sequence of PMAIP1 is 5′-GGAAGUCGAGUGUGCUACU-3′. The transfection of siRNA was following the manufacturer’s protocol of X-tremeGENE Transfection Reagent (Roche Molecular Biochemicals, Mannheim, Germany). Cells were seeded in 6-well plates and transfected with control or target siRNA on the second day. Cells were treated with indicated concentration of PTL for another 24 hours and harvested for Western blot analysis or Annexin V assay.

### Apoptosis assay

Apoptosis was evaluated using Annexin V-FITC/PI apoptosis detection kit purchased from BIO-BOX Biotech (Nanjing, China) following the manufacturer’s instructions. Briefly, 2×10^6^cells were harvested and washed twice with pre-cold PBS and then resuspended in 500 μl binding buffer. 5 μl of annexin V-FITC and 5 μl of Propidium Iodide (PI) were added to each sample and then incubated at room temperature in dark for 10 minutes. Analysis was performed by FACScan flow cytometer (Becton Dickinson, San Jose, CA).

## Results

### Parthenolide effectively inhibits the growth of human lung cancer cells through induction of apoptosis and cell cycle arrest

**Figure 1:**
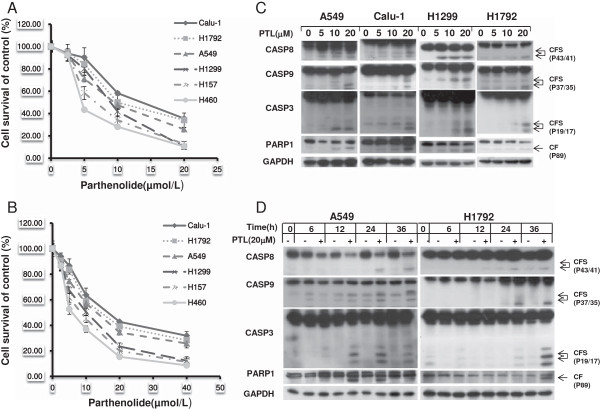
**Parthenolide inhibits cell growth (A, B) and induces apoptosis in a concentration-dependent (C) and a time-dependent manner (D).** The indicated cell lines were seeded in 96-well plates and treated with the given concentration of PTL for 48 hrs. Cell survival was estimated using SRB assay **(A)** and MTT assay **(B)**. Points: mean of four replicate determinations; bars: S.D. The indicated cells were treated with indicated concentrations of PTL for 24 hrs **(C)** or treated with 20 μmol/L PTL for various lengths of time and harvested for Western blot analysis **(D)**. CF: cleaved form.

It has been reported that parthenolide has antitumor effects on various cancer cells. Hence, we examined the inhibition effect of PTL on human NSCLC cells by treating the cells with various concentrations for 48 h and then conducting SRB and MTT assay. As is shown, PTL had a dose-dependent growth inhibition effect on NSCLC cells Calu-1, H1792, A549, H1299, H157, and H460 (Figure 1A, B). To characterize the mechanism by which PTL induces growth inhibition in human NSCLC cells, we first determined the effect of PTL on induction of apoptosis by western blot analysis. The data showed that PTL could induce cleavage of apoptotic proteins such as CASP8, CASP9, CASP3 and PARP1 both in concentration- and time-dependent manner in tested lung cancer cells, indicating that apoptosis was trigged after PTL exposure (Figure 1C, D). In addition to induction of apoptosis, PTL also induced G_0_/ G_1_ cell cycle arrest in a concentration- dependent manner in A549 cells and G_2_/M cell cycle arrest in H1792 cells (Additional file 1: Figure S1). The difference in cell cycle arrest induced in these two cell lines may be due to the p53 status [37,38]. Collectively, these results show that PTL inhibits the growth of human lung cancer cells through induction of apoptosis and/or cell-cycle arrest.

### Parthenolide triggers extrinsic apoptosis by up-regulation of TNFRSF10B expression

**Figure 2:**
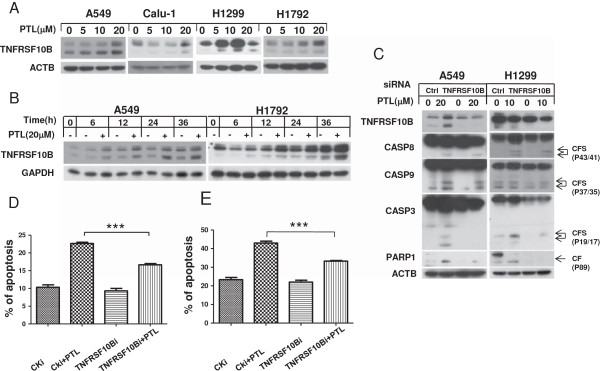
**Parthenolide induces extrinsic apoptosis by up-regulate TNFRSF10B in a dose-dependent (A) and a time-dependent (B) manner, and inhibiting TNFRSF10B expression by siRNA decreases parthenolide–induced apoptosis (C, D and E).** The indicated cells were treated with indicated concentrations of PTL for 24 hrs **(A)** or treated with 20 μmol/L PTL for various lengths of time and harvested for Western blot analysis **(B)**. A549 **(C, D)** and H1299 **(C, E)** cells were seeded in 6-well plates and on the second day transfected with control or TNFRSF10B siRNA. A549 cells were treated with 20 μmol/L PTL while H1299 cells with 10 μmol/L for another 24 hours after 48 hrs of transfection and harvested for Western blot analysis **(C)** or for detection of apoptotic cells using Annexin V/PI staining **(D, E)**. Points:mean of three replicate determinations; bars: S.D. P value < 0.05.

In order to understand the molecular mechanism of PTL-induced apoptosis in NSCLC cell lines, several apoptosis-related proteins were examined. Data showed that TNFRSF10B was up-regulated after exposure to PTL (Figure 2A, B). After TNFRSF10B expression was knocked down using siRNA method, the cleavage of CASP8, CASP9, CASP3 and PARP1 induced by PTL treatment were receded compared with control siRNA knockdown (Figure 2C). The analysis of Annexin V staining showed that apoptosis was inhibited when TNFRSF10B was knocked down (Figure 2D, E). It can be concluded that PTL up-regulates TNFRSF10B and contributes to apoptosis induction in lung cancer cells.

### CFLAR is down-regulated in parthenolide -induced apoptosis

**Figure 3:**
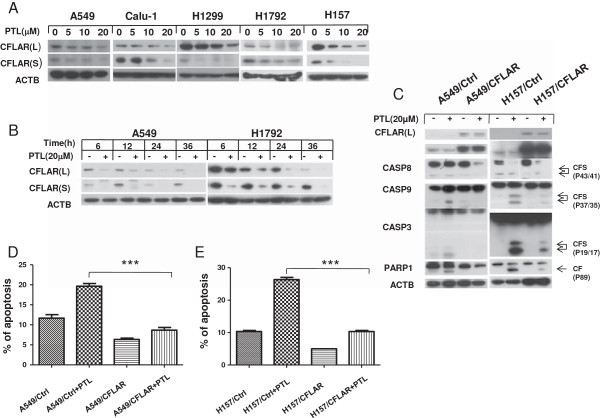
**CFLAR is down-regulated in parthenolide -induced apoptosis in a dose-dependent (A) and a time-dependent (B) manner, and overexpression of CFLAR**_**L **_**can protect cells from parthenolide-induced apoptosis (C,D and E).** The indicated cells were treated with indicated concentrations of PTL for 24 hrs **(A)** or treated with 20 μmol/L PTL for various lengths of time and harvested for Western blot analysis **(B)**. Indicated cells were seeded in 6-well plates and on the second day treated with 20 μmol/L PTL for another 24 hours and harvested for Western blot analysis **(C)** or for detection of apoptotic cells using Annexin V/PI staining **(D, E)**. Points:mean of three replicate determinations; bars: S.D. P value < 0.05.

Since CFLAR is an important modulator of extrinsic apoptotic pathway, we also detected the levels of CFLAR and found that both CFLAR_L_ (Long form) and CFLAR_S_ (Short form) were down-regulated in a concentration- and time-dependent manner after PTL treatment (Figure 3A, B). Compared with control cells, cleavage of pro-caspases and PARP1 were weaker in A549/CFLAR_L_ cells which over-expressing CFLAR_L_ (Figure 3C). Annexin V staining showed PTL induced less apoptosis in A549/CFLAR_L_ cells than that in control cells (Figure 3D). We got same results in H157/CFLAR_L_ cells (Figure 3C, E). This implicated that CFLAR_L_ could prevent human lung cancer cells from apoptosis induced by PTL treatment. Therefore, we can summarize that TNFRSF10B and CFLAR_L_ are involved in PTL-induced extrinsic apoptosis.

### PMAIP1 and MCL1 contribute to parthenolide -induced intrinsic apoptosis

**Figure 4:**
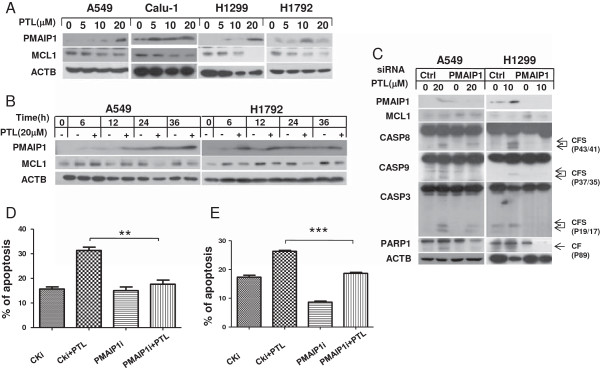
**Parthenolide induces intrinsic apoptosis through up-regulating PMAIP1 expression and down-regulating MCL1 level in a dose-dependent (A) and a time-dependent (B) manner, and knockdown of TNFRSF10B by siRNA decreases parthenolide–induced apoptosis (C, D and E).** The indicated cells were treated with indicated concentrations of PTL for 24 hrs **(A)** or treated with 20 μmol/L PTL for various lengths of time and harvested for Western blot analysis **(B)**. A549 **(C, D)** and H1299 **(C, E)** cells were seeded in 6-well plates and on the second day transfected with control or PMAIP1 siRNA. A549 cells were treated with 20 μmol/L PTL while H1299 cells with 10 μmol/L for 24 hours after 48hs of transfection and harvested for Western blot analysis **(C)** or for detection of apoptotic cells using Annexin V/PI staining **(D, E)**. Points:mean of three replicate determinations; bars: S.D. P value < 0.05.

We wonder if PTL could also activate intrinsic apoptotic pathway in lung cancer cells. Since PMAIP1 and MCL1 are both important proteins in intrinsic signaling pathway, we detected their expression after PTL treatment. Western blot analysis revealed that MCL1 was decreased in both concentration- and time-dependent manners after PTL exposure, while PMAIP1 was up-regulated (Figure 4A, B). Gene silencing experiment presented that when PMAIP1 was knocked down, the expression of MCL1 was partially increased and the cleavage of pro-caspases and PARP1 induced by PTL were reduced (Figure 4C). Annexin V staining analysis showed that apoptosis induced by PTL was weakened after knocking down of PMAIP1 (Figure 4D, E). It could be concluded that the intrinsic apoptosis process induced by PTL is through PMAIP1 and MCL1 axis.

### Parthenolide induces apoptosis through activation of ER stress response

**Figure 5:**
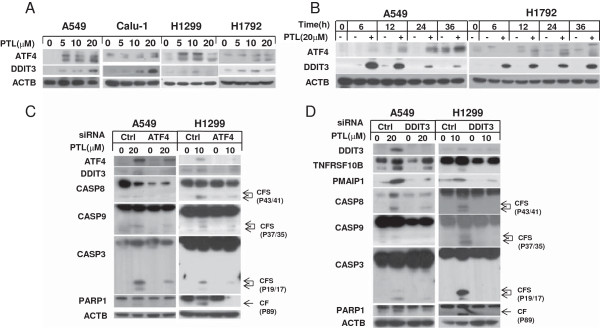
**Parthenolide induces apoptosis through up-regulating ATF4 and DDIT3 in a dose-dependent (A) and a time-dependent (B) manner, and knockdown of ATF4 by siRNA decreases parthenolide–induced DDIT3 and apoptosis (C). Knockdown of DDIT3 decreases parthenolide-induced TNFRSF10B, PMAIP1 expression and apoptosis (D).** The indicated cells were treated with indicated concentrations of PTL for 24 hrs **(A)** or treated with 20 μmol/L PTL for various lengths of time and harvested for Western blot analysis **(B)**. A549 **(C, D)** and H1299 **(C, D)** cells were seeded in 6-well plates and on the second day transfected with control or ATF4 **(C)** or DDIT3 **(D)** siRNA. A549 cells were treated with 20 μmol/L PTL while H1299 cells with 10 μmol/L for 24 hours after 48 hrs of transfection and harvested for Western blot analysis.

**Figure 6:**
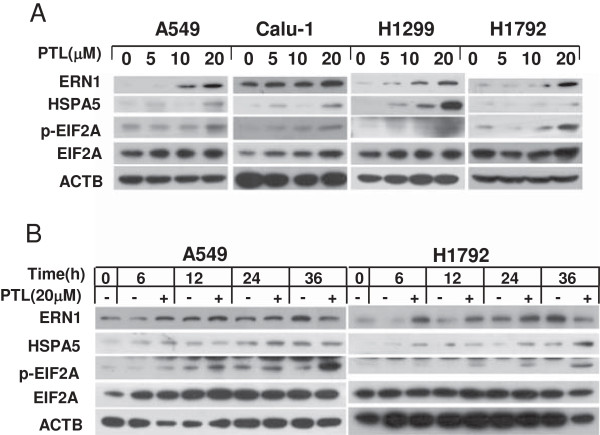
**Parthenolide up-regulates endoplasmic reticulum hallmarks ERN1, HSPA5 and p-EIF2A in a dose-dependent (A) and a time-dependent (B) manner.** The indicated cells were treated with indicated concentrations of PTL for 24 hrs **(A)** or treated with 20 μmol/L PTL for various lengths of time and harvested for Western blot analysis **(B)**.

DDIT3, which is a target protein of ATF4, is reported to regulate the expression of TNFRSF10B and PMAIP1 by binding to their promoter sites [27]. Therefore, we wonder if PTL induces TNFRSF10B and PMAIP1 through ATF4-DDIT3 axis. We examined expression of ATF4 and DDIT3 after PTL treatment. Western blot revealed that PTL could up-regulate ATF4 and DDIT3 in both concentration- and time-dependent manner (Figure 5A, B). When ATF4 was knocked down, DDIT3 was decreased, and activation of pro-caspases was weakened at the same time compared with control knockdown cells (Figure 5C). In addition, apoptosis was suppressed when DDIT3 was knocked down, while the expression of TNFRSF10B and PMAIP1 were decreased simultaneously (Figure 5D). Since ATF4 and DDIT3 are important hallmarks involved in ER stress pathway, we examined the expression of other molecules in ER stress signaling such as ERN1, HSPA5 and p-EIF2A as well [39]. We found that they were both increased after PTL treatment (Figure 6A, B). All these data indicated that PTL induces apoptosis through activation of ER stress response.

### Parthenolide selectively eradicates lung cancer stem-like cells

**Figure 7:**
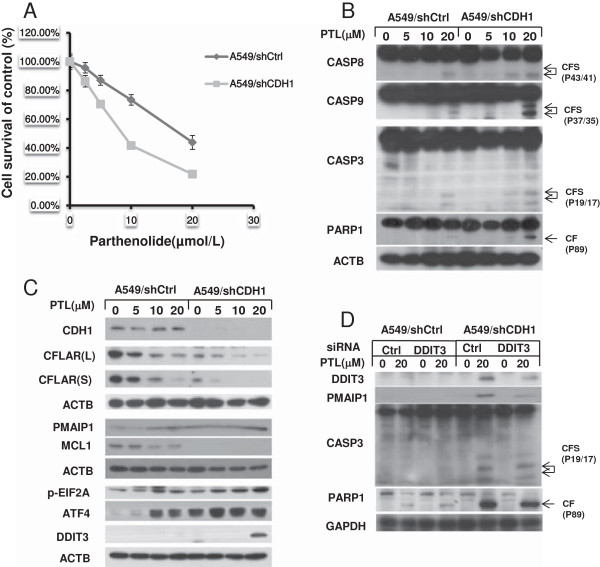
**Parthenolide selectively inhibits cell growth (A) and induces stronger apoptosis (B) in A549/shCDH1 cells and apoptosis, and ER stress related proteins are up-regulated more clearly by parthenolide in A549/shCDH1 cells than that in control cells (C). Knockdown of DDIT3 decreases parthenolide–induced PMAIP1 and apoptosis (D).** The indicated cell lines were seeded in 96-well plates and treated with the given concentration of PTL for 24 hrs **(A)**. Live cell number was estimated using SRB assay for calculation of cell survival. Points: mean of four replicate determinations; bars: S.D. A549/shCtrl and A549 shCDH1 cells were treated with indicated concentrations of PTL for 24 hrs. Both attached and suspended cells were harvested for Western blot analysis; CF: cleaved form **(B,C)**. A549/shCtrl and A549 shCDH1 cells were seeded in 6-well plates and on the second day transfected with control or DDIT3 siRNA. Cells were treated with 20 μmol/L PTL for 24 hours after 48 hrs of transfection and harvested for Western blot analysis **(D)**.

Weinberg et al. has demonstrated that knocking down of CDH1/E-cadherin with shRNA could make the cells have stem-like properties [40]. We had demonstrated that A549/shCDH1 cells in which CDH1/E-cadherin expression is inhibited had stronger capacity of proliferation, migration and invasiveness [32]. Furthermore, we found that the expression of SOX2 and POU5F1 which were considered to be the makers of stem cells were up-regulated in A549/shCDH1 cells (Additional file 1: Figure S2) [41,42]. So in order to determine why PTL could selectively eradicate cancer stem-like cells, A549/shCDH1 cell line was used to mimic cancer stem cells and the A549/shCtrl cell line served as control. SRB assay showed PTL was more effective in inhibiting the growth of A549/shCDH1 cells than that of A549/shCtrl cells (Figure 7A). Western blot data showed that PTL could induce stronger cleavage of pro-caspases and PARP1 in A549/shCDH1 cell line (Figure 7B), which means that PTL could trigger stronger apoptosis in A549/shCDH1 cells compared with control cells. Furthermore, apoptosis-related proteins were detected in A549/shCtrl and A549/shCDH1 cells side by side. Both long form and short form of CFLAR levels were down-regulated even more clearly in A549/shCDH1 cells than that in control cells after PTL treatment. We also found that MCL1 was reduced more dramatically in A549/shCDH1 cells, while PMAIP1 was up-regulated on contrary after PTL treatment compared with the control cells (Figure 7C). Taken together, we conclude that both extrinsic apoptosis and intrinsic apoptosis induced by PTL are enhanced in A549/shCDH1 cells. The levels of p-EIF2A, ATF4 and DDIT3 were also examined. Data showed that their expression was further up-regulated in A549/shCDH1 cells after PTL treatment compared with A549/shCtrl cells (Figure 7C). DDIT3 was knocked down in the two cell lines simultaneously, and PMAIP1 was down-regulated and apoptosis was receded (Figure 7D). Therefore, we propose that the reason why PTL has a selective effect towards A549/shCDH1 cells is because PTL somehow triggers much more intensive ER stress response in cancer stem-like cells and further enhances the expression of ATF4 and DDIT3, leading to up-regulation of PMAIP1 and eventually, the induction of apoptosis.

## Discussion

Parthenolide, a sesquiterpene lactone used for therapy of inflammation, has been reported to have anti-cancer property. Significantly, recent studies revealed PTL could selectively eradicate acute myelogenous leukemia stem cells and breast cancer stem-like cells, but the molecular mechanism is still unknown. In our study, we found that PTL can induce apoptosis in NSCLC cells in both concentration- and time-dependent manner. In addition, PTL could also induce G_0_/ G_1_ cell cycle arrest in A549 cells and G_2_/M arrest in H1792 cell line. The possible reason to this difference may be is that p53 in A549 cells is wide type while it is mutant in H1792 cell. However, in all tested cell lines, PTL induces obvious apoptosis no matter what the p53 status is.

Subsequently, we detected apoptosis-related proteins and found TNFRSF10B was up-regulated after PTL treatment. TNFRSF10B Knockdown resulted in subdued activation of caspases and apoptosis. Results also showed that CFLAR was decreased after exposed to PTL. Over-expressing ectopic CFLAR_L_ can weaken the cleavage of caspases and apoptosis induced by PTL. We conclude that both TNFRSF10B and CFLAR are responsible for PTL-induced extrinsic apoptotic pathway.

Proteins involved in intrinsic apoptotic pathway were also examined in our research. MCL1 was found to be down-regulated under PTL treatment, while PMAIP1 was increased on contrary. PMAIP1 Knockdown resulted in increased level of MCL1 and weakened cleavage of caspases and apoptosis. To summarize, the apoptosis induced by PTL in lung cancer cells is via both intrinsic and extrinsic apoptotic pathways, the intrinsic apoptosis is mediated through PMAIP1/MCL1 axis.

**Figure 8:**
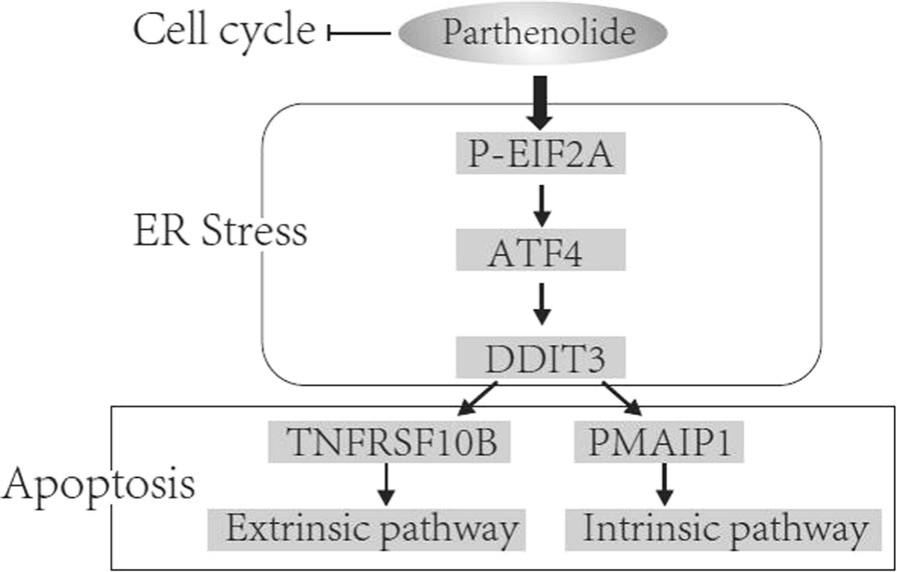
**Summary of parthenolide-induced signaling pathway in NSCLC cell lines.** Briefly, PTL induces ER stress response and eventually results in up-regulation of DDIT3 which could increase the expression of TNFRSF10B and PMAIP1 by binding to their promoter sites as a transcription factor. As the critical members of extrinsic and intrinsic apoptotic pathway respectively, TNFRSF10B and PMAIP1 consequently activate these two pathways to induce apoptosis in human lung cancer cells.

We and others have reported that DDIT3 could up-regulate the expression of TNFRSF10B and PMAIP1 [36,43], so we examined DDIT3 expression in PTL-induced apoptosis. Results showed that DDIT3 was up-regulated by PTL, and DDIT3 knockdown resulted in reduced expression of TNFRSF10B and PMAIP1 which leading to weaker apoptosis compared with control. DDIT3 is an important molecule in ER stress pathway. We next analyzed whether PTL could induce ER stress. ERN1, HSPA5, p-EIF2A and ATF4, which are all key proteins involved in ER stress, were all up-regulated by PTL in both concentration- and time-manner. ATF4 Knockdown also led to DDIT3 reduction and weaker apoptosis. All these results indicated that PTL can induce apoptosis in lung cancer cells via activation of ER stress response (Figure 8). PTL is reported to induce ROS which can trigger ER stress response [44]. It was found that the NAC could protect cell form PTL induced apoptosis, which is the scavenging agent of ROS [7]. But whether PTL triggers ER stress through ROS in our system requires future study.

What interested us most is how PTL selectively kills cancer stem cell. The cells in which CDH1 expression is inhibited can present properties of cancer stem cells [32,40]. We found that the expression of stem cell maker SOX2 and POU5F1/Oct-4 were up-regulated in A549/shCDH1 cells. So, we used A549/shCDH1 cells to explore the apoptosis induced by PTL in cancer stem cells. Major proteins related in PTL-induced signal pathway were detected. We observed that the level of TNFRSF10B was increased, and CFLAR was decreased more clearly in A549/shCDH1 cells compared with A549/Ctrl cells after PTL treatment, which could explain the enhanced cleavage of CASP8. Furthermore, MCL1 level was much lower, and PMAIP1 level was much higher in A549/shCDH1 cells than that in control cells after PTL exposure. Although the basal levels of p-EIF2A in the two cell lines were almost equal, it was up-regulated more clearly in A549/shCDH1 cells than that in control cells after PTL treatment. In addition, ATF4 and DDIT3 were both up-regulated in A549/shCDH1 cells more dramatically than that in control cells after exposure with PTL. Afterwards, we knocked down DDIT3 in the two cell lines side by side and found that PMAIP1 was down-regulated, and apoptosis was receded. We propose that the reason why PTL has a selective effect towards cancer stem-like cells is that PTL somehow induced stronger ER stress response and further enhances the expression of ATF4 and DDIT3, which leads to up-regulation of PMAIP1 and eventually, the apoptosis induction in cancer stem-like cells.

In summary, our work demonstrates that parthenolide induces both extrinsic and intrinsic apoptosis via ER stress signaling pathway in human NSCLC cells (Figure 8). Moreover, parthenolide induces stronger ER stress and apoptosis in cancer stem-like cells which may account for its selective effect in apoptosis induction. Collectively, this study provides important mechanistic insight into potential cancer treatment with parthenolide as well as our understanding for cancer stem cells.

## Competing interests

The authors declare that they have no competing interests.

## Authors’ contributions

LS and XL designed research; XZ and LS performed research; XZ and LS analyzed data; XZ, XL and LS wrote the paper. All authors read and approved the final manuscript.

## Supplementary Material

Additional file 1: Figure S1 Parthenolide induces cell cycle arrest in NSCLC cell lines. A549 (A) and H1792 (B) cells were treated with different concentrations of PTL for 24 hours. After treatment, the cells were harvested for cell cycle assays. **Figure S2.** Cancer stem cell makers are up-regulated in A549/shCDH1 cells. The expression level of SOX2 and POU5F1 were detected in A549/shCtrl and A549/shCDH1 cells by Western Blot assay. Click here for file
