# Supplementary figures and images for: Parthenolide induces apoptosis via TNFRSF10B and PMAIP1 pathways in human lung cancer cells
Source: J Exp Clin Cancer Res. 2014 Jan 6;33(1):3. doi: 10.1186/1756-9966-33-3 (PMC3892099; doi:10.1186/1756-9966-33-3)

## Slide 1
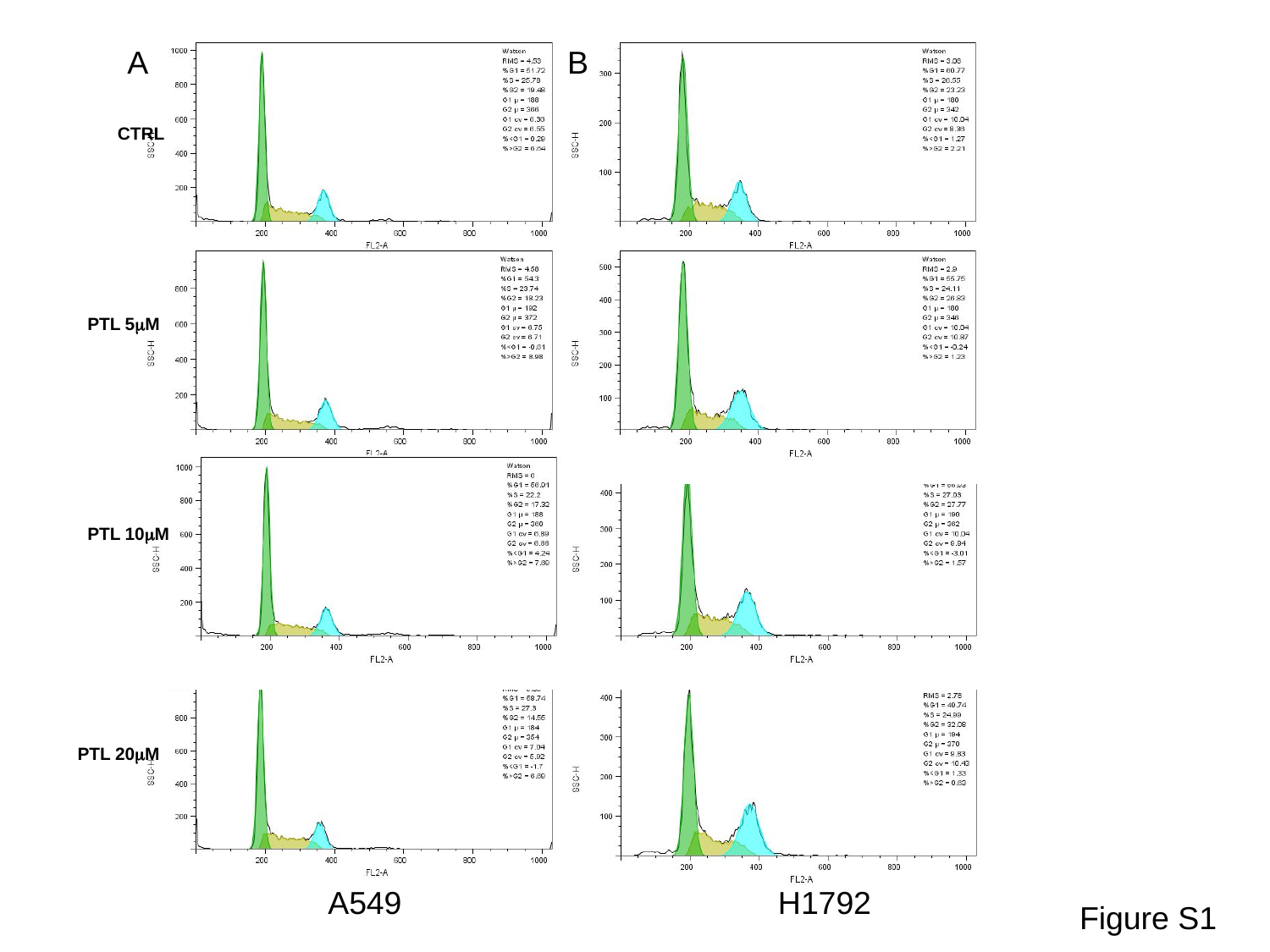

A
B
CTRL
PTL 5μM
PTL 10μM
PTL 20μM
A549
H1792
Figure S1

## Slide 2
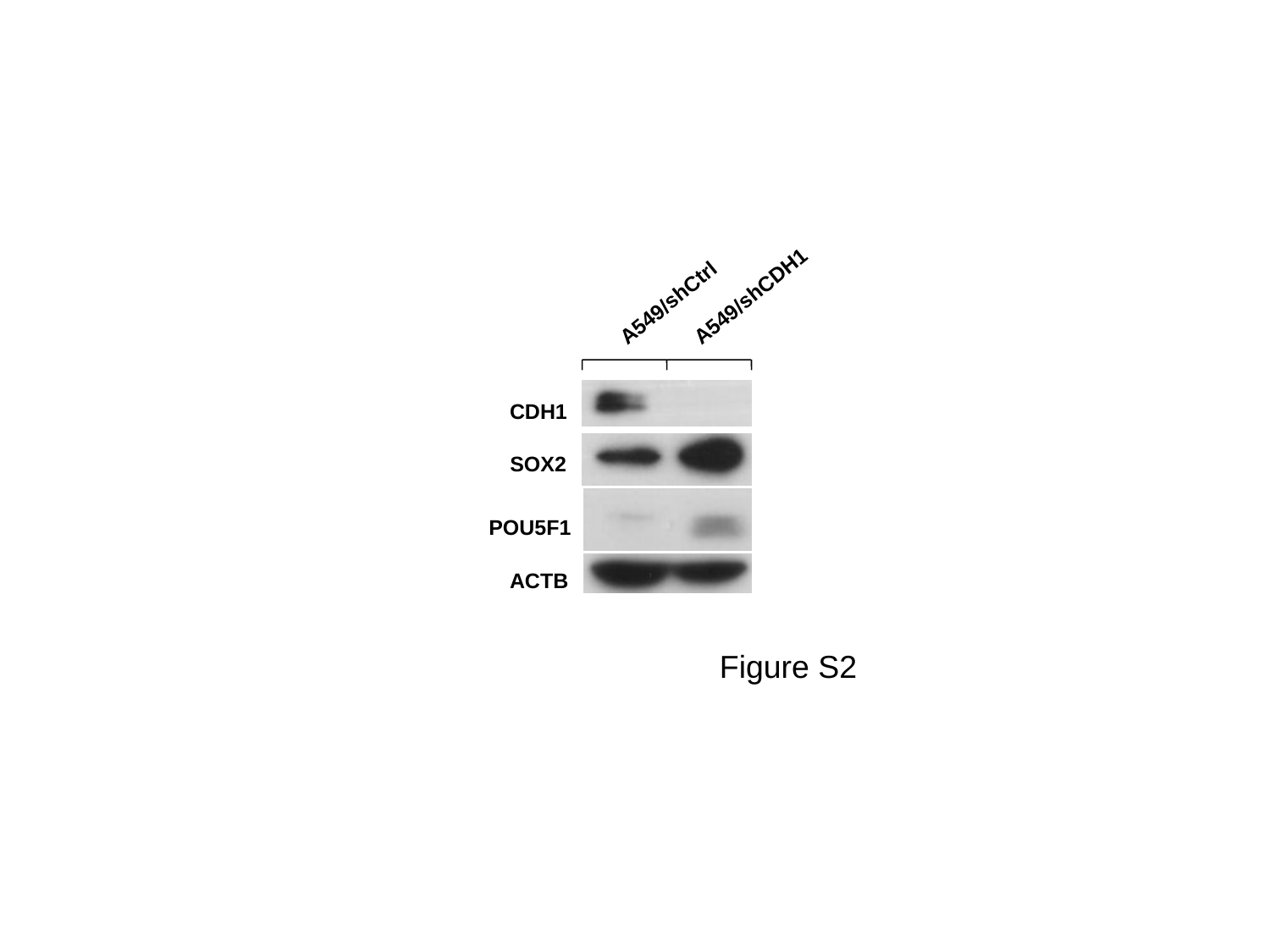

A549/shCtrl
A549/shCDH1
CDH1
SOX2
POU5F1
ACTB
Figure S2

Supplement: Additional file 1: Figure S1 — Parthenolide induces cell cycle arrest in NSCLC cell lines. A549 (A) and H1792 (B) cells were treated with different concentrations of PTL for 24 hours. After treatment, the cells were harvested for cell cycle assays. Figure S2. Cancer stem cell makers are up-regulated in A549/shCDH1 cells. The expression level of SOX2 and POU5F1 were detected in A549/shCtrl and A549/shCDH1 cells by Western Blot assay. [file 1756-9966-33-3-S1.ppt]
